# Prevalence of opportunistic infections in Syrian inflammatory bowel disease patients on biologic therapy: a multi-center retrospective cross-sectional study

**DOI:** 10.1186/s12879-025-11063-6

**Published:** 2025-05-04

**Authors:** Marouf Alhalabi, Hussam Aldeen Alshiekh, Shadi Alsaiad, Mouayad Zarzar

**Affiliations:** 1 Gastroenterologist at Gastroenterology Department of Damascus Hospital, Almujtahed Street, Damascus, Syria; 2 Gastroenterology Department of Ibn Al-Nafees Hospital, Damascus, Syria

**Keywords:** Ulcerative colitis, Crohn's disease, Inflammatory bowel disease, Hepatitis B, Hepatitis C, Tuberculosis, Cytomegalovirus

## Abstract

**Background:**

Hepatitis B, hepatitis C, cytomegalovirus (CMV), and tuberculosis (TB) pose significant risks to patients with inflammatory bowel disease (IBD) receiving biological therapy. However, data on the prevalence of these infections in Syria are scarce.

**Methods:**

We conducted a retrospective chart review of IBD patients receiving biologic therapy at Damascus Hospital and Ibn Al-Nafees Hospital, two major public institutions in Syria, between January 2021 and November 2024. A minimum sample size of 130 was estimated; however, all available records were reviewed.

**Results:**

Among 185 IBD patients (104 from Damascus and 81 from Ibn Al-Nafees), 51.4% had ulcerative colitis and 47.6% had Crohn’s disease. The smoking prevalence was 9.2%, which was higher in Crohn’s disease (5.9%) than in ulcerative colitis (3.2%). TST performed in 61.1% of patients, with 4.3% positivity, and interferon-gamma release assay (IGRA) in 8.7% (1.1% positive). Hepatitis B surface antigen (HBsAg) and anti-HBc antibodies were found in 2.7% and 5.4% of the patients, respectively, while hepatitis C seroprevalence was low (0.5%). CMV seropositivity was high in Damascus (50.8%), with two cases (1.1%) of CMV colitis. Biologic therapies included infliximab (42.7%), ustekinumab (24.3%), golimumab (10.8%), and adalimumab (6.5%). Data gaps, particularly in viral serology and TB screening, are notable.

**Conclusion:**

This study identifies deficiencies in TB/hepatitis B screening (notably anti-HBs Ab) and elevated CMV seroprevalence among Syrian IBD patients receiving biologics, extending to immunosuppressed cohorts (rheumatology, dermatology, oncology). Insufficient screening heightens occult infection/reactivation risks, necessitating standardized pretreatment protocols to reduce morbidity in high-risk populations.

**Clinical trial number:**

Not applicable.

**Supplementary Information:**

The online version contains supplementary material available at 10.1186/s12879-025-11063-6.

## Background

Chronic infections like hepatitis B (HBV), hepatitis C (HCV), cytomegalovirus (CMV), and tuberculosis (TB) continue to pose major global public health challenges. HBV and HCV in particular are among the leading causes of preventable deaths due to chronic liver disease [1–4]. Current global data suggest that around 5.8% of the population carries hepatitis B surface antigen (HBsAg), indicating chronic HBV infection, while approximately 10.3% test positive for HCV antibodies, reflecting past or present exposure to the virus [1]. When TB coexists with these conditions—especially in high-risk populations—the burden on both patients and healthcare systems intensifies. In Syria, years of conflict have devastated the healthcare system. Routine vaccination programs have been disrupted, millions have been displaced, and access to medical care is patchy at best. This combination of overcrowding, malnutrition, and fragmented care increases the risk of infectious disease transmission. Yet, most global guidelines are based on research from high-income, stable countries and don’t fully address the realities faced in conflict zones. While there’s a lack of precise data on TB prevalence in Syria—partly due to ongoing instability—the widespread destruction of healthcare infrastructure and massive displacement have clearly raised the population’s vulnerability to TB, as well as to HBV and HCV [5]. What’s needed are context-specific guidelines that take into account the diagnostic challenges and heightened risk factors in such settings, like cramped shelters and limited laboratory access. CMV infection is another key issue. Globally, CMV seroprevalence ranges from 45% to nearly 100%, with the highest rates seen in developing countries [6], however, in Syria and much of the Middle East, the extent of CMV exposure is still under-researched. Factors like poor sanitation and limited screening options only add to the risk [7]. In immunocompromised populations, including patients with inflammatory bowel disease (IBD) receiving biologics, CMV colitis presents a complex clinical challenge, increasing the risk of colectomy and complicating disease management [8]. In immunocompromised individuals—such as patients with inflammatory bowel disease (IBD) who are receiving biologic therapies—CMV can reactivate and lead to colitis. This complicates treatment and increases the risk of needing a colectomy [8]. Similarly, co-infections with HBV, HCV, or TB in IBD patients add another layer of diagnostic and therapeutic complexity, highlighting the importance of thorough screening and surveillance. Syria presents a unique epidemiological landscape—where endemic infections intersect with conflict-driven healthcare inequalities and broken systems of care. These realities offer crucial insights for improving international guidelines so they better reflect the needs of vulnerable populations. Addressing these gaps is essential to improving infection control, treatment protocols, and healthcare resource allocation in areas marked by displacement, poverty, and limited access to care. This study aims to assess the prevalence of HBV, HCV, CMV, and TB among immunosuppressed IBD patients in Syria, with the goal of informing more tailored strategies to manage and reduce co-infection-related complications and deaths.

## Methods

### Study design

#### Diagnostic criteria

IBD subtypes (UC and CD) were identified using standardized clinical, endoscopic, and histological criteria based on ECCO guidelines [9, 10]. Hepatitis B virus (HBV) infection was defined as HBsAg seropositivity, whereas hepatitis C virus (HCV) infection required anti-HCV positive, which was validated by tests meeting AASLD and EASL standards [11–14]. Latent tuberculosis (TB) was diagnosed by a tuberculin skin test (TST; induration ≥ 5 mm) or an interferon-gamma release assay (IGRA), but active TB needed microbiological or clinical-radiographic confirmation [15]. CMV colitis was confirmed in colonic tissue using histology or PCR [16–18].

### Sample size and sampling technique

**Fig. 1:**
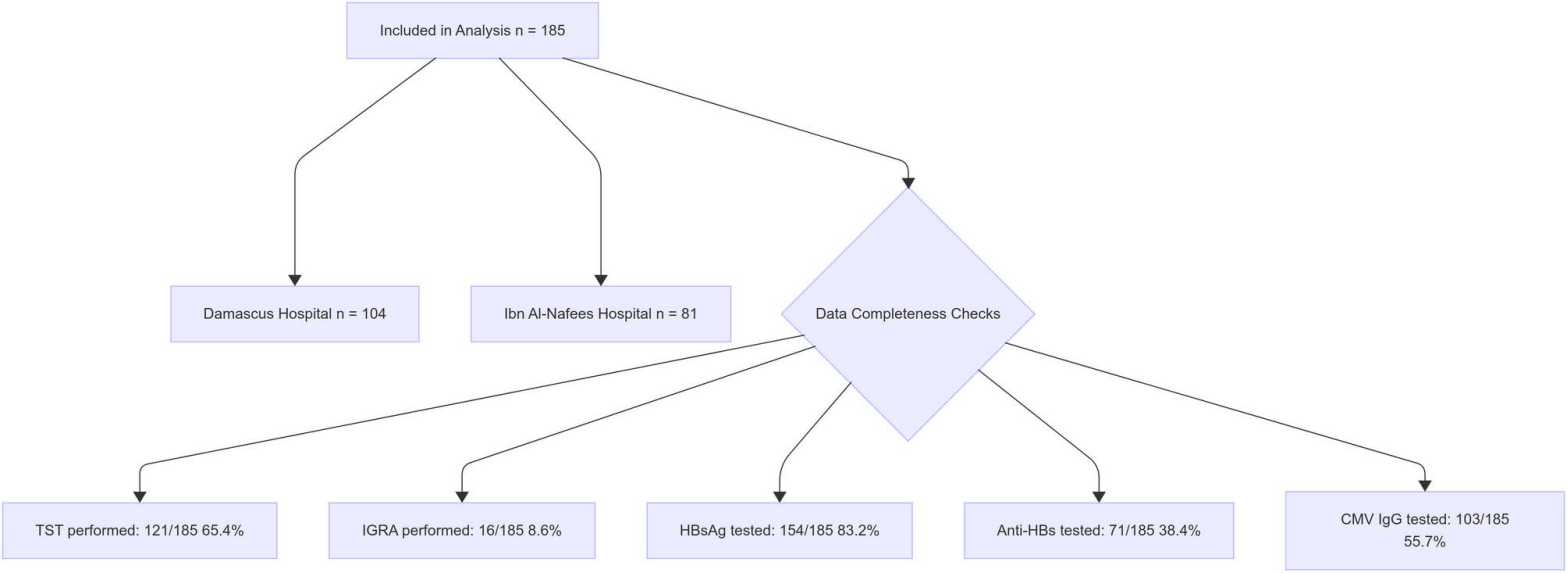
Flowchart of screening process for IBD patients on biologic therapy

The sample size (n) was obtained using the proportional equation: [n = Z^2^ × P× (1 − P)/d^2^] [19], proposed the following hypothesis: Z = 1.96 at the 95% confidence level, with a margin of error (d) of 0.05. Initial calculations used published seroprevalences: CMV IgG (P = 0.909) [20]. HBV (P = 0.056 [21].and HCV (P = 0.028 [22]. Yielding minimum targets of 128 (CMV), 82 (HBV), and 42 (HCV) patients. Despite recruitment challenges in a conflict-affected setting, all accessible records (n = 185) from 2021–2024 were reviewed to optimize statistical power and minimize selection bias. Notably, the observed HCV seroprevalence (0.5%) was lower than anticipated, resulting in wide confidence intervals and reduced precision for HCV estimates. A retrospective analysis of medical records was performed at Damascus Hospital and Ibn Al-Nafees Hospital, two prominent public healthcare institutions in Damascus, Syria, affiliated with the Ministry of Health. The objective was to determine the prevalence of hepatitis B virus (HBV), hepatitis C virus (HCV), cytomegalovirus (CMV), and tuberculosis (TB) infections among patients diagnosed with inflammatory bowel disease (IBD). Inclusion criteria for this study required patients to have a confirmed diagnosis of IBD based on established clinical, endoscopic, and/or histological criteria as defined by relevant guidelines [9, 10]; (2) Documentation of treatment at the IBD Unit included verified evidence of patients receiving medical management for inflammatory bowel disease within the specified unit. To enhance precision, we focused on analyzing records of all patients who underwent immunosuppressive therapy. The largest sample size was for CMV seroprevalence which was initially calculated as n = 123, based on a 95% confidence interval, a 5% margin of error, and an assumed prevalence of 90.9%. All available records (n = 185) were reviewed to optimize statistical power, minimize selection bias, and account for potential missing data—common challenges in retrospective conflict-zone studies. The observed HCV seroprevalence was 0.5%, which was significantly lower than the 2.8% prevalence used in the initial sample size estimate. Although the study sample of 185 exceeded the projected objective of 42 for HCV, the low prevalence resulted in a limited number of positive cases, decreasing the accuracy of the HCV seroprevalence estimate and providing broader confidence ranges. This limitation may impair the study’s ability to robustly characterize HCV seroprevalence in the target population. Figure 1 summarizes the study’s design and participant selection process [23]. A total of 185 IBD patients on immunosuppressive medication were retrospectively enrolled at Damascus Hospital and Ibn Al-Nafees Hospital (2021–2024). Eligibility required a confirmed IBD diagnosis based on ECCO criteria [9, 10] and verified immunosuppressive therapy. Despite initial sample size targets of 128 for CMV, 82 for HBV, and 42 for HCV, recruitment hurdles in conflict-affected Syria limited enrollment to 185 patients. The flowchart divides data collecting areas (demographics, serological testing, and tuberculosis screening) and standardization processes to reduce inter-hospital variability. Missing data, resulting from diagnostic reagent shortages and institutional resource constraints during the humanitarian crisis, were rigorously labeled as ‘unknown’ to reduce bias.

### Instruments and data collection

Following a thorough literature analysis, a structured questionnaire (Supplementary File S1) was created to fit with the study’s aims. The instrument, which was originally written in English, was revised for cultural and linguistic significance. A qualified physician extracted data from medical records (January 2021–November 2024), including demographics (age, sex), medical history (comorbidities, surgeries), pharmacological history (biologics, immunosuppressants), IBD subtype (UC/CD), disease activity indices, hepatitis serology (HBsAg, anti-HBc, anti-HBs, anti-HCV), and mycobacterial testing (TST/IGRA). Anti-HBs levels were used as a proxy for HBV immunity, however distinguishing between vaccine-derived and infection-acquired immunity was not possible.

### Data extraction and standardization

A pilot-tested questionnaire with established criteria (e.g., TST is positive at ≥ 5 mm in immunocompromised patients, ≥ 10 mm in high-risk individuals, and ≥ 15 mm in low-risk patients) ensured consistency between Damascus and Ibn Al-Nafees hospitals. Research assistants were trained together to standardize term interpretation (for example, CMV colitis requires histopathology/PCR) and data entry. Ambiguous factors, such as active smoking (≥ 1 cigarette/day for ≥ 6 months), were defined beforehand. A senior researcher assessed 20% of randomly selected records each site and resolved differences through consensus. Identical assays (CMV IgG ELISA, HBsAg ECLIA) were used, and missing data was classified as “unknown”. Inter-rater reliability for crucial variables (IBD subtype and TST interpretation) was near-perfect (κ = 0.81, 95% CI: 0.73–0.89).

### Statistical tests

R software was used for prevalence estimates (with 95% confidence intervals). Subgroup comparisons (hospital, IBD subtype, smoking status) employed χ² or Fisher’s exact tests, with 95% CI [24].

### Missing data handling

Missing TST and anti-HBs findings were omitted from the primary analysis to avoid misclassification. Sensitivity analysis compared the best-case (missing = negative) and worst-case (missing = positive) scenarios. Systemic gaps in testing, caused by reagent shortages (e.g., tuberculin), prioritizing of acute IBD care, and institutional cost barriers (e.g., CMV IgG exclusion), mirrored conflict-related resource constraints during Syria’s humanitarian crisis (2012–2024). These constraints most likely underestimated latent infection prevalence, underscoring the importance of context-specific methods in crisis circumstances.

### Data security

Participants’ files were allocated unique identities and kept in secured cabinets. Data was entered into a password-protected Microsoft Access database with specified fields to reduce errors. De-identified records will be electronically archived for three years after the study, with access limited to approved people.

## Results

Demographic and clinical characteristics of the cross-sectional study are summarized in Table 1. While age and sex distributions were comparable between hospitals, significant institutional disparities emerged in smoking prevalence and immunosuppressive therapy utilization. Notably, biologic agent prescribing patterns varied, reflecting differences in local protocols. These findings underscore the heterogeneous healthcare practices across centers, likely exacerbated by resource constraints during Syria’s humanitarian crisis. Statistical comparisons accounted for confounders, with methodological rigor adhering to STROBE guidelines.

**Table 1:** Demographic and clinical characteristics

Variable	Damascus Hospital (*n* = 104)	Ibn Al-Nafees Hospital (*n* = 81)	Pooled Cohort (*n* = 185)	*p*-value
**Age (years)**,** median (IQR)**	34 (24.75–43)	31 (22.75–40)	34 (24–43)	*p* = 0.12
**Female Sex**,** n (%) [95% CI]**	58 (55.8%) [46.0–65.2%]	42 (51.9%) [41.0–62.6%]	100 (54.1%) [46.8–61.2%]	*p* = 0.60
**IBD Subtype**,** n (%) [95% CI]**				*p* = 0.91
- Ulcerative Colitis (UC)	53 (51.0%) [41.4–60.5%]	42 (51.9%) [40.9–62.6%]	95 (51.4%) [44.1–58.6%]	
- Crohn’s Disease (CD)	51 (49.0%) [39.5–58.6%]	37 (45.7%) [35.0–56.7%]	88 (47.6%) [40.4–54.9%]	
**Active Smoking**,** n (%) [95% CI]**	17 (16.3%) [10.3–24.7%]	0 (0%) [0–5.1%]	17 (9.2%) [5.7–14.3%]	*p* < 0.001
**Immunosuppressive Therapies**				
- Azathioprine, n (%) [95% CI]	80 (76.9%) [68.0–84.0%]	74 (91.4%) [83.3–95.8%]	154 (83.2%) [77.1–88.1%]	*p* = 0.006
- Infliximab, n (%) [95% CI]	51 (49.0%) [39.5–58.6%]	28 (34.6%) [25.0–45.6%]	79 (42.7%) [35.7–50.0%]	*p* = 0.06
- Ustekinumab, n (%) [95% CI]	25 (24.0%) [16.9–32.9%]	20 (24.7%) [16.7–35.0%]	45 (24.3%) [18.7–30.9%]	*p* = 0.91

Infection screening outcomes and prevalence are detailed in Table 2. Marked institutional variability was observed in tuberculosis and cytomegalovirus testing, with significant gaps in hepatitis B immunity surveillance. The near-universal CMV seropositivity at one hospital contrasted starkly with the absence of testing at the other, highlighting systemic inequities. These results emphasize the urgent need for standardized pre-biologic screening protocols in conflict settings, where diagnostic resource shortages disproportionately impact vulnerable populations.

**Table 2:** Infection screening and prevalence

Variable	Damascus Hospital (*n* = 104)	Ibn Al-Nafees Hospital (*n* = 81)	Pooled Cohort (*n* = 185)	*p*-value
**Tuberculosis (TST)**				
- Performed, n (%) [95% CI]	80 (76.9%) [68.0–84.0%]	40 (49.4%) [38.6–60.2%]	120 (64.9%) [57.6–71.5%]	*p* < 0.001
- Positive, n/N (%) [95% CI]	7/80 (8.8%) [4.8–15.2%]	1/40 (1.2%) [0.03–6.7%]	8/120 (4.3%) [2.2–8.3%]	*p* = 0.06
- UC vs. CD, n/N (%) [95% CI]	3/53 (5.7%) [1.9–15.1%] vs. 4/51 (7.8%) [3.1–18.3%]	0/42 (0%) [0–8.4%] vs. 1/37 (2.7%) [0.5–13.9%]	3/95 (3.2%) [1.1–8.9%] vs. 5/88 (5.7%) [2.4–12.7%]	*p* = 0.47
**Hepatitis B (HBsAg)**				
- Tested, n (%) [95% CI]	87 (83.7%) [75.3–89.6%]	66 (81.5%) [71.4–88.6%]	153 (82.7%) [76.5–87.6%]	*p* = 0.70
- Positive, n/N (%) [95% CI]	4/87 (4.6%) [1.8–11.1%]	1/66 (1.5%) [0.3–8.2%]	5/153 (3.3%) [1.4–7.4%]	*p* = 0.38
- Smokers vs. Non-Smokers, n/N (%) [95% CI]	2/17 (11.8%) [3.3–34.3%]	-	2/17 vs. 3/168 (1.8%) [0.6–5.1%]	*p* = 0.09
**Cytomegalovirus (CMV)**				
- IgG Tested, n (%) [95% CI]	103 (99.0%) [94.7–99.8%]	0 (0%) [0–5.1%]	103 (55.7%) [48.3–62.8%]	< 0.001
- IgG Positive, n/N (%) [95% CI]	94/103 (91.3%) [84.4–95.4%]	0/0 (N/A)	94/103 (91.3%) [84.4–95.4%]†	*p* < 0.001
- UC vs. CD, n/N (%) [95% CI]	47/53 (88.7%) [77.5–94.8%] vs. 47/51 (92.2%) [81.5–97.0%]	-	47/95 (49.5%) [39.6–59.4%] vs. 47/88 (53.4%) [42.9–63.7%]	*p* = 0.57
**Hepatitis C (Anti-HCV)**				
- Tested, n (%) [95% CI]	89 (85.6%) [77.5–91.1%]	58 (71.6%) [60.7–80.5%]	147 (79.5%) [72.9–84.8%]	*p* = 0.02
- Positive, n/N (%) [95% CI]	1/89 (1.1%) [0.2–6.0%]	0/58 (0%) [0–6.2%]	1/147 (0.7%) [0.1–3.7%]	*p* = 1.00

### Impact of missing data

TST prevalence estimates ranged from 4.3% (8/185; 95% CI: 1.4–7.2%) to 38.9% (72/185; 95% CI: 31.9–45.9%), while anti-HBs seropositivity ranged from 6.5% (12/185; 95% CI: 3.0–10.0%) to 68.1% (126/185; 95% CI: 61.4–74.8%). These extremes, shown in broad confidence ranges, confirm that observed rates are driven by incomplete screening rather than biological prevalence, underscoring the importance of standardized techniques to reduce diagnostic uncertainty. The broad confidence intervals in sensitivity analyses, especially for worst-case scenarios, indicate the significant uncertainty caused by missing data, emphasizing the limits of retroactive conflict-zone studies.

## Discussion

Biologic medicines pose a particular danger of reactivating latent infections such as hepatitis B virus (HBV) and tuberculosis (TB), both of which can cause significant morbidity and even death [8]. Hepatitis B reactivation (HBVr) is the resurgence of viral replication in patients with prior or chronic HBV infection, caused by the persistence of covalently closed circular DNA (cccDNA) in hepatocytes despite apparent virological remission; under immunosuppression, cccDNA can resume transcription and generate new virions [24, 25]. Although diligent prophylaxis and surveillance can prevent most HBVr episodes, the concern remains in everyday practice [25, 26]. Although HBVr is preventable with appropriate management, it remains a concern in clinical practice [12, 14, 27]. There is consensus that HBVr is defined by an increase in serum HBV DNA among previously exposed persons, however the precise criteria vary. The Asian Pacific Association for the Study of the Liver (APASL) defines reactivation as a ≥ 2-log rise in HBV DNA or an absolute level greater than 100 IU/mL when no baseline is available. In contrast, the American Association for the Study of Liver Diseases (AASLD) uses stratified cut-offs according to hepatitis B surface antigen (HBsAg) status and initial viral load, and separately characterizes “hepatitis flare” by elevated alanine aminotransferase (ALT), a distinction not made by APASL [14, 27]. These disparate frameworks need individualized guideline selection in clinical decision-making. Risk stratification differs between societies. Both APASL and the American Gastroenterological Association (AGA) agree that prolonged high-dose corticosteroids (> 20 mg prednisone equivalent for > 4 weeks) and anti-TNF agents (adalimumab, infliximab, golimumab) have an HBVr risk of more than 10%; however, APASL classifies these therapies as high-risk, while AGA classifies them as moderate-risk. Ustekinumab is consistently deemed moderate-risk (1–10%), while medicines such as methotrexate, azathioprine, or short low-dose steroids remain low-risk [28–30]. A recent meta-analysis found HBVr rates above 10% among chronic HBV carriers receiving adalimumab, infliximab, or ustekinumab—findings that fit more closely with APASL than AGA guidelines and reflect the scarcity of previous evidence on cytokine inhibitors [31]. The absence of consistent HBVr definitions across worldwide standards impedes cross-study comparisons and obscures genuine incidence estimates, emphasizing the urgent need for a uniform reactivation criterion [14, 27]. To minimize HBVr, three main antiviral methods are used: (1) Prophylactic therapy started before immunosuppression [8, 14, 27, 28]; (2) Pre-emptive therapy initiated upon early virological markers but prior to full reactivation [12, 27, 28, 32]; and (3) On-demand therapy reserved for confirmed reactivation based on clinical and laboratory criteria [14]. Selection is based on baseline serology, the strength and duration of immunosuppression, and patient-specific variables. Notably, our sample revealed low rates of anti-HBs testing, highlighting the necessity of baseline vaccination and establishing anti-HBs titers > 10 IU/L in inflammatory bowel disease (IBD) patients lacking prior immunity [8, 12, 14, 27]. Data on direct-acting antivirals (DAAs) in IBD patients co-infected with hepatitis C remains limited to case series, and the effect of immunosuppressive agents on sustained virologic response is unclear; nonetheless, existing guidelines advocate HCV treatment according to standard national protocols, with close monitoring for IBD flares during DAA administration [8].Similarly, immunosuppression increases the chance of latent tuberculosis infection (LTBI) reactivation in IBD, which often leads to more severe disease progression. Pre-treatment LTBI screening—ideally before any immunosuppressive exposure—is crucial, with repeat evaluation warranted if therapy changes or in high-risk circumstances [8, 33, 34]. A network meta-analysis reveals that biologic exposure increases total tuberculosis risk, which includes both new infections and reactivations [35]. Ustekinumab appears to carry a modest TB reactivation risk. In psoriasis studies and large cohorts, no cases occurred among prophylaxed LTBI patients [36–41]. Although rare instances (e.g., a pediatric Crohn’s case on combo therapy) indicate that prolonged use may still bring risks [42, 43]. Conventional immunomodulators (azathioprine, methotrexate, ciclosporin) and short-term corticosteroids pose no major TB risk, making routine LTBI treatment unnecessary in most situations [44, 45]. Annual rescreening is advisable for individuals in endemic regions or with ongoing exposure [46]. To evaluate LTBI, consider epidemiological risk, clinical examination, chest radiography, and interferon-γ release assays (IGRA) or tuberculin skin tests (TST). Immunosuppressive drugs can reduce TST and IGRA sensitivity, thus testing at IBD diagnosis—before immunosuppression—is ideal [47, 48]. A TST induration ≥ 5 mm indicates a positive result, however specificity is low (about 5% of immunocompetent positives advance to active TB) and may be influenced by remote BCG vaccination [49–51]. LTBI treatment must be completed before initiating biologics; if active IBD requires urgent therapy, a minimum four-week overlap after LTBI treatment onset is recommended [8]. In this restricted sample, ustekinumab revealed a reassuring safety profile among patients with either positive TST or evidence of prior HBV exposure, regardless of the preventive strategy [40]. While TST and IGRA remain crucial for LTBI screening, their poor sensitivity in immunocompromised individuals requires an integrated strategy that includes epidemiological risk assessment, clinical evaluation, and immunological testing to maximize tuberculosis risk management [52]. In our Damascus Hospital sample, anti-CMV IgG was identified in 91.3% of patients, while CMV colitis developed in just 1.9%. This aligns with recent observations that, whereas CMV exposure is nearly universal in IBD, clinically severe colitis is unusual [53]. The lack of comparable data from Ibn Al-Nafees Hospital prevents direct inter-center comparison and emphasizes the importance of coordinated data collection to understand CMV prevalence and its clinical consequences across institutions [54]. Despite the retrospective methodology and conflict-related healthcare disruptions that preclude direct comparison to contemporaneous general population data, our findings are consistent with regional patterns: HBV and HCV prevalence among biologic candidates was lower than that reported among blood donors, perhaps reflecting preselection through comorbidities screening, but CMV seroprevalence approximated endemic Middle Eastern exposure [22, 55–58]. The difference between our TST positivity rate (4.3%) and the estimated latent TB prevalence in Syria (10–15%) demonstrates the influence of immunosuppression and poor screening on diagnostic yield [59–61] HBV, transferred by percutaneous or mucosal exposure, poses a substantial reactivation risk under biologic therapy, potentially leading to fulminant hepatitis if unchecked [62–64]. while TNF-α inhibitors predispose to TB reactivation and dissemination [65, 66]. CMV exploits intestinal inflammation in IBD to reactivate, exacerbating colitis severity and increasing colectomy ris [58]. Although our study focused on HBV, HCV, TB, and CMV, endemic infections such as HPV—prevalent in conflict zones and linked in neoplastic progression—require consideration in future research [67]. Despite Syria’s high HBV newborn immunization coverage (≥ 85%) and ubiquitous BCG delivery (≥ 90%), anti-HBs seropositivity was only 9.6% in our group, suggesting attenuated vaccine responses in immunosuppressed people [68, 69]. Universal BCG hampers TST interpretation by raising false-positive rates, although IGRA specificity is unchanged. International recommendations advocate HBV revaccination and prevention in seronegative individuals starting biologics [70, 71]. Significant gaps in pre-biologic screening—61.6% missing anti-HBs, 20.5% untested for HCV, 44.3% missing CMV IgG, and 34.6% missing TST—raise the risk of undiscovered infections and negative consequences. We recommend for standardized screening bundles (HBsAg, anti-HBc, anti-HBs, HCV serology, CMV IgG, and dual TST/IGRA testing) and preventative measures, such as isoniazid for LTBI and antiviral therapy in accordance with AASLD guidelines. Our retrospective design and humanitarian setting present many limitations, including selection bias from incomplete data at Ibn Al-Nafees (100% CMV IgG and 91.4% IGRA missing), information bias due to non-standardized documentation (61.5% anti-HBs missing), and the inability to demonstrate causality. Screening techniques vary by institution (e.g., CMV IgG testing in 99% at Damascus against none at Ibn Al-Nafees; TST completion rates of 76.9% versus 49.4%, *p* < 0.001), limiting comparability. Conflict-related diagnostic reagent shortages, inconsistent power supply, and fragmented health systems most likely contributed to non-random missingness and the emphasis of acute care over latent infection screening. Screening techniques vary by institution (e.g., CMV IgG testing in 99% at Damascus against none at Ibn Al-Nafees; TST completion rates of 76.9% versus 49.4%, *p* < 0.001), limiting comparability. Conflict-related diagnostic reagent shortages, inconsistent power supply, and fragmented health systems most likely contributed to non-random missingness and the emphasis of acute care over latent infection screening [7, 55, 56, 72–76].

Nevertheless, sensitivity analyses that account for missing data support our basic finding that observed hazards are driven by incomplete screening rather than true infection prevalence. Prospective studies using established techniques, mixed-effects modeling, and multiple imputation are required to validate these findings and inform guidance for conflict-affected countries.

## Conclusions

In this first Middle Eastern study, opportunistic infection screening in IBD patients on biologics was inadequate, with anti-HBs testing skipped in 61.5% and tuberculosis evaluation insufficient in 34.6%. These gaps highlight the importance of consistent AGA/AASLD guideline-driven screening to prevent reactivation. The lack of CMV and HBV data at Ibn Al-Nafees Hospital highlights structural inequities in war zones, necessitating integrated regional surveillance. Future research should validate algorithms in resource-limited settings, use cost-effective diagnostics (e.g., combined TST/IGRA), standardize HBV-reactivation criteria, and evaluate ustekinumab safety in latent TB/HBV. These findings also apply to immunomodulator-treated rheumatoid arthritis, dermatological, and cancer patients.

## Electronic supplementary material

Below is the link to the electronic supplementary material.

Supplementary Material 1

Supplementary Material 2

## Data Availability

The data that support the findings of this study are available on request from the corresponding author, [MH]. The data are not publicly available due to containing information that could compromise the privacy of research participants.

## Abbreviations

UC: Ulcerative colitis
CD: Crohn’s disease
HBV: hepatitis B
HCV: Hepatitis C
CMV: Cytomegalovirus
TB: Tuberculosis
HbsAg: Hepatitis B surface antigen
Anti-HCV: Anti-hepatitis C virus
TST: Mantoux tuberculin skin tests
IGRA: Interferon-Gamma Release Assays
Anti-HBc: Hepatitis B core antibody
anti-HbsAB: Hepatitis B Surface Antibody
ECCO: European Crohn’s and Colitis Organisation
ACG: American College of Gastroenterology
IBD: Inflammatory bowel disease
PCR: Polymerase chain reaction
TNFα: Tumour Necrosis Factor alpha
