# Supplementary material for: Prevalence of opportunistic infections in Syrian inflammatory bowel disease patients on biologic therapy: a multi-center retrospective cross-sectional study
Source: BMC Infect Dis. 2025 May 4;25:652. doi: 10.1186/s12879-025-11063-6 (PMC12051298; doi:10.1186/s12879-025-11063-6)
Supplement: Supplementary file 2 — Supplementary Material 2 [file 12879_2025_11063_MOESM2_ESM.pdf]

IBD & Opportunistic infections

☐ **Smoker** **State** ☐ F ☐ M **SEX** **DOB** **Name**

Bio treatment date **HBV Diagnosis date** ☐ CD ☐ UC **IBD**

☐ Not Studied ☐ Negative ☐ Positive **IGRA** ☐ Not Studied ☐ Negative ☐ Positive **TST**

TB treatment if reactivaiton ☐ **TB Reactivaiton**

Treatment **HCV-PCR** ☐ Not Studied ☐ Negative ☐ Positive **Anti-HCV**

Treatment ☐ **CMV-Colitis** ☐ Not Studied ☐ Negative ☐ Positive **Anti-CMV**

☐ Not Studied ☐ Negative ☐ Positive **Anti-HBc** ☐ Not Studied ☐ Negative ☐ Positive **HBsAg**

☐ Not Studied ☐ Negative ☐ Positive **HBeAg** ☐ Not Studied ☐ Negative ☐ Positive **Anti-HBs**

☐ Not Studied ☐ Negative ☐ Positive **Anti-HBe** **Treatment** **HBV-PCR**

Date ☐ **ALF** **Date** ☐ **Acute HBV** **Date** ☐ **HBVr**

Age

☐ L4:isolated upper disease ☐ L3:ileocolonic ☐ L2:colonic ☐ L1: ileal **CD classification**

☐ p perianal disease ☐ B3 penetrating ☐ B2 stricturing ☐ B1 non-stricturing, non-penetrating

☐ Extensive UC ☐ Left sided UC ☐ E1Ulcerative proctitis **UC classification**

☐ S3 ☐ S2 ☐ S1 ☐ S0

☐ ımunodulator loss of response ☐ stula/Diseaes ☐ Steroid resistance ☐ steroid dependent **Biology indication**

Date ☐ **AZA** **Date** ☐ **Steroides** **Treatment**

Date ☐ **GOLI** **Date** ☐ **ADA** **Date** ☐ **IFX**

Date ☐ **UST**

Tests

Cr **INR** **BIL** **HGB** **AST** **ALT**

Other information

S0: Asymptomatic

S1: Mild UC: Passage of four or fewer stools/day (with or without blood), absence of any systemic illness, and normal inflammatory markers (ESR)

S2: Moderate UC: Passage of more than four stools per day but with minimal signs of systemic toxicity

S3: Severe UC: Passage of at least six bloody stools daily, pulse rate of at least 90 beats per minute, temperature of at least 37.5°C, haemoglobin of less than 10.5 g/100 ml, and ESR of at least 30 mm/h
